# What’s Left of the Left–Right Dimension? Why the Economic Policy Positions of Europeans Do Not Fit the Left–Right Dimension

**DOI:** 10.1007/s11205-017-1575-7

**Published:** 2017-02-08

**Authors:** Simon Otjes

**Affiliations:** Documentation Centre Dutch Political Parties, Groningen University, Broerstraat 4, 9712 CP Groningen, The Netherlands

**Keywords:** Economic issues, Public opinion, Elite-mass linkage, Socialisation, Left–right dimension

## Abstract

In political science the economic left–right dimension plays a central role. A growing body of evidence shows that the economic policy preferences of a large segment of citizens do not scale sufficiently. Using Mokken scale analysis, this study determines the causes of this phenomenon. Differences in the extent to which the economic policy preferences of citizens fit the left–right dimension can be explained in terms of the interaction between individual level and political system-level variables: citizens who spend more attention to politicians with views that conform to the left–right dimension, have views that conform to the left–right dimension. There is also a role for the legacy of communist dictatorship: citizens who were socialised in democratic countries have views that fit the left–right dimension better than those socialised during communism.

## Introduction

The economic left–right dimension plays a central role in models of political behaviour and democratic representation (Costello et al. 2012; Downs 1957; Kriesi et al. 2008; Mair 2007; Thomassen 1999). The central idea is that citizens who favour a more egalitarian distribution of income also favour more government intervention in the economy. A growing body of evidence suggests that citizens’ economic policy preferences cannot be summarised in terms of a single left–right dimension. For one, citizens’ opinions on the welfare state are often ideologically inconsistent and contradictory (Achterberg et al. 2011; Derks 2004, 2006; De Koster et al. 2013; Goerres and Prinzen 2011; Roosma et al. 2012; Roosma et al. 2014). Other research has shown that economic interventionism and egalitarianism (Otjes 2014) or support for equal opportunities and equal outcomes (Fossati and Häusermann 2014) do not constitute a single dimension. A number of studies have noted that citizens’ views on economic issues do not cohere, but these studies have treated this as a methodological or a case-specific issue (e.g. Costello et al. 2012; Dolezal et al. 2013; Sperber 2010; Wagner and Kritzinger 2012; Walczak et al. 2012). It appears that citizens’ opinions on economic issues deviate from the left–right dimension that plays such a crucial role in models of political behaviour and democratic representation.

This phenomenon merits better understanding, so this article will address two closely related questions. First this article will analyse *whether the economic policy preferences of European citizens can be summarised in a single left*–*right dimension*. In this sense, this study echoes the classic study by Converse (1964), which found that citizens in general do not have policy positions that fit the left–right dimension. But this study will delve further into this phenomenon by examining its causes, by asking *under what conditions citizens have economic policy preferences that do not fit the economic left*–*right dimension*. Here the article builds on classic work on political socialisation (Key 1961; Neundorf 2009; Zaller 1992) by arguing that the structure of voter preferences is the result of an interaction between the political system and the individual voters. On the one hand, polity-level characteristics, such as a history of communism and the structure of elite competition, shape voters’ preferences. On the other hand, only voters raised under communism are affected by its legacy, and only voters who pay attention to politics are likely to be affected by what political elites do. By showing that there are meaningful differences between regions and groups of voters in the extent to which citizens’ views fit the left–right dimension, this article lends credence to the idea that this phenomenon is not some kind of measurement error, but reflects meaningful differences between citizens.

This article has the following structure. First, this article will describe the academic debate about the value of the left–right dimension for understanding voters’ economic preferences. Then this article will offer a number of explanations for why voters’ views on economic issues may not fit the economic left–right dimension. Next, this article will discuss how those explanations were tested. Mokken scaling analysis (Mokken 1971) was used to explore the coherence of citizens’ economic preferences. This article first looks at aggregate scaling results to determine whether citizens’ views on economic issues collectively can be considered as a single economic left–right scale; then it looks at individual-level results, using a measure of person-fit as the dependent variable. This measure, known as Guttman errors (Meijer 1994), expresses the extent to which individual citizens’ answering patterns fit the tested model. Finally, the relevance of the results for future research will be discussed.

## The Economic Left–Right Dimension and Challenges to This Dimension

The left–right dimension plays a central role in theoretical and empirical studies of democratic politics (Mair 2007; Thomassen 1999). The economic left–right dimension is thought of as the centrepiece of a left–right ‘super-issue’ that also orders citizens’ views on environmental and ethical issues (Inglehart 1984). The extent to which the left–right dimension suffices to understand voters’ positions is a matter of lively debate (Evans et al. 1996; Kriesi et al. 2008; Van der Brug and Van Spanje 2009).

What has been contested far less is the notion that there is a single economic dimension, that structures citizens’ opinions on economic issues. This concerns at least two issues: economic interventionism and economic egalitarianism. First is the classic Downsian question: ‘[h]ow much government intervention in the economy should there be?’ (Downs 1957, p. 116). Right-wing voters tend to support *laissez*-*faire* economic principles, while left-wing voters tend to support an active government. Economic egalitarianism is the second element. Lipset et al. (1954, p. 1135) proposed that egalitarianism is the core of the left–right dimension: ‘[b]y left we shall mean advocating social change in the direction of greater equality—political, economic or social; by right we shall mean (…) opposing change towards greater equality’. The right believes that inequalities reward good performance, while the left favours a more equal redistribution of resources. In Bobbio’s (1996) influential model, the division between equality and inequality forms the core of the difference between left and right. Noëll and Thérien (2008) posited that the right and the left have different conceptions of equality: the left favours equal outcomes and the right favours equal opportunities.

As mentioned above, there is growing evidence that citizens do not think about economic matters in these dichotomous terms. The first line of evidence comes from studies of opinions on the welfare state. Achterberg et al. (2011, p. 1) observed that ‘the international research literature generally understands economic egalitarianism, the traditional leftist quest for economic equality and redistributive policies and support for the welfare state as two closely related phenomena that can be measured by highly interchangeable scales’. A number of quantitative studies have shown that this is not the case (Achterberg et al. 2011; De Koster et al. 2013; Derks 2004, 2006; Roosma et al. 2012, 2014). Thus, supporting the principle of egalitarianism is not the same as supporting the welfare state.^1^ Goerres and Prinzen (2011) used focus groups to examine the items traditionally used to measure citizens’ positions about welfare state policies. They, too, found that citizens’ preferences were not ideologically consistent.

A number of political science studies have examined the extent to which voters’ views fit the left–right dimension from a theoretical perspective. Otjes (2014) established that economic egalitarianism and economic interventionism, the two core elements of the left–right dimension, are not necessarily the same. Fossati and Häusermann (2014) found that support for equal opportunities and equal outcomes are not opposing positions on the same dimension, as Noëll and Thérien (2008) proposed, but rather support for equal opportunities and equal outcomes represent two independent dimensions. These studies were the first to look at this phenomenon as a theoretical challenge to the prevalence of the left–right dimension in political science. Other studies have observed the failure of economic left–right scales but treated it as a methodological or case-specific issue (e.g. Costello et al. 2012; Sperber 2010; Wagner and Kritzinger 2012; Walczak et al. 2012).

Some students of public opinion may not be surprised that citizens’ views on economic matters do not reflect the left–right dimension. Converse (1964, p. 245) already observed that in the United States ‘a large proportion of an electorate simply does not have meaningful beliefs’, not even about those questions that have been heavily debated in the political and public spheres. The lack of fit to the left–right dimension has been observed in other countries as well (Butler and Stokes 1974; Dolezal et al. 2013). Zaller (1992, p. 93) argued that citizens do not have ‘true attitudes’ but rather express ideologically inconsistent views under different conditions. This study re-examines these findings and delves further into the causes of this phenomenon.

## Why Citizens’ Views on Economic Matters May Deviate from the Left–Right Dimension

The section above sketched the debate about the extent to which voters’ views on economic matters are actually structured by the left–right dimension. This section will offer a number of explanations for why voters’ views may deviate from a left–right pattern, building on the classic work in the field of political socialisation (Key 1961; Zaller 1992). The explanations focus on the interaction between characteristics of individual voters and their political context.

### The Political Debate in Democratic Systems

Elite-level and mass-level political spaces are not necessarily identical (Van der Brug and Van Spanje 2009). Still, the structure of party competition may be an important determinant of citizens’ economic policy positions: people take cues from politicians about how to think about political issues (Key 1961; Neundorf 2009).

For this mechanism to work, two conditions must be met. First, voters should pay attention to politics. Second, the cues that politicians give should reflect the left–right dimension. A large proportion of voters does not pay attention to political debates about economic issues (Bartels 2008). The political elite is unlikely to shape the views of those who are not interested in politics, as they do not pay enough attention to the political debate to be influenced by it (Luskin 1990). Citizens are more likely to understand political issues in the terms of the political elite when those citizens listen to what politicians have to say.

Second, the nature of elite cues matters. If politicians talk in terms of a single left–right dimension, citizens are more likely to think in those terms (Neundorf 2009). If politicians express preferences that are less consistent with the left–right dimension, citizens are likely to develop economic policy positions that also do not fit the left–right dimension. The nature of party competition shapes the extent to which voters’ views fit the left–right dimension. Combined, the expectations for voter-level political interest and elite-level cues lead to the following hypothesis:

1. Democratic Debate Hypothesis: the economic policy preferences of politically interested citizens in political systems where politicians have economic policy preferences that fit the left–right dimension are more likely to fit the left–right dimension than the economic policy preferences of politically disinterested citizens in those political systems.

### The Legacy of Authoritarian Systems

The Democratic Debate Hypothesis depends on the actual existence of elite conflict. In a dictatorship, where there is no elite conflict, citizens do not learn to think in opposing political terms (Mondak and Gearing 1998). While democratic political systems have been firmly established in Western Europe for many decades, new democracies were formed in Central and Eastern Europe only in the early 1990s, after decades of communist rule. Mondak and Gearing (1998) showed that the experience of dictatorship in Central and Eastern Europe alienated citizens from politics: people who were socialised during the dictatorship are less interested in politics and less likely to understand economic interests in political terms than their counterparts from Western democracies. The underlying mechanism is socialisation: voters are socialised to think in terms of the elite. If there is no elite conflict, citizens are not socialised to think in left–right terms about policy issues.

But this is not just a regional difference; it is a generational difference within these post-communist systems. Only older citizens in those systems are likely to be affected by the legacy of dictatorship. Citizens who were socialised after their countries democratised are more likely to have economic policy preferences that correspond to the economic left–right dimension than citizens who were socialised during the communist dictatorship. This expectation can be summarised in the following hypothesis.

2. Authoritarian Legacy Hypothesis: the economic policy preferences of citizens who were more recently socialised in former dictatorships are more likely to fit the left–right dimension than the economic policy preferences of citizens who were socialised earlier in those countries.

## Methodology

This section concerns the methodological approach of this article. First, it will discuss the chosen scaling method (Mokken scaling). Second, it will discuss the dependent variable of this study, Guttman errors, which reflect the extent to which a voter’s view fits a Mokken scale. Third, it will discuss the independent variables in the study. The 2014 European Election Survey is analysed (Schmitt et al. 2015), which had at least 1000 respondents in every member state of the European Union in 2014 (except for Malta and Luxembourg, where the sample size was at least 500). The total response rate was 65% (TNS 2015). In total, there are 30,064 cases in the data set. Due to missing variables, this was reduced to 22,876 in the analysis.

### Mokken Scaling

This article uses Mokken scaling to test the extent to which voters’ opinions fit the left–right dimension (Mokken 1971), because it is a simple and intuitive measure of respondent fit. It allows one to differentiate between citizens whose opinions about economic matters fit the left–right dimension to a greater or lesser extent.

Mokken scaling was developed to assess the quality of educational tests (Mokken 1971). It tests the assumption that a set of items can be ordered on a continuum from simplest to most difficult: that is, items for which the largest number of respondents gave the correct answer to those for which the fewest gave the correct answer.

Since this article uses eleven-step items concerning economic statements instead of binary data, polytomous Mokken scaling is used (Van der Ark 2007). This scaling method assesses the extent to which the item steps (instead of items) can be ordered, from those to which most respondents tended to give left-wing answers to those to which most respondents tended to give right-wing answers. If these item steps cannot be ordered as such, this indicates that citizens’ views on these issues do not follow a left–right pattern. Mokken scaling cannot handle missing values, so cases with missing values were deleted list-wise. Alternatives such as Cronbach’s α are based on a number of assumptions about the data, for instance that voter positions follow a normal distribution (Van Schuur and Kiers 1994). In contrast, Mokken scaling makes minimal assumptions about data distribution.

Mokken scaling is closely related to the Guttman scale; it is a non-parametric, probabilistic version of this scale (Van Schuur 2003). A key concept in understanding Mokken scales is Guttman errors. This is the number of item steps that respondents that answer the ‘difficult’ items correctly (or the most right-wing items affirmatively) answer the easy items incorrectly (or the fewest right-wing items negatively). The Guttman error for polytomous items for respondent k is defined as:

1 $$G_{k} = \mathop \sum \limits_{i = 1}^{I - 1} \mathop \sum \limits_{j = i + 1}^{I} w_{ij} f_{ijk}$$

Here I is the total number of item steps, and i and j are item steps that are ordered from ‘easy’ to ‘difficult’. f_ij_ can either be zero, if the ordered pattern is not violated, or one if it is. These violations are weighted by w_ij_, the number of previously missed item steps (Zijlstra et al. 2007, pp. 7–8).^2^

One can aggregate the number of Guttman errors made by all respondents in the H-value. This compares the number of Guttman errors to a pattern where the items are independent of each other (Mokken 1971). H-values can also be calculated for specific items (the H_i_-values) and the relationship between pairs of items (the H_ij_-values). The formulae for polytomous H-, H_i_- and H_ij_-values are listed below.

2 $$H_{ij} = 1 - \frac{{\mathop \sum \nolimits_{k = 1}^{K} w_{ij} f_{ijk} }}{{F_{ij} }}$$

3 $$H_{i} = 1 - \frac{{\mathop \sum \nolimits_{j = 1, j \ne i}^{I} \mathop \sum \nolimits_{k = 1}^{K} w_{ij} f_{ijk} }}{{\mathop \sum \nolimits_{j = 1, j \ne i}^{I} F_{ij} }}$$

4 $$H = 1 - \frac{{\mathop \sum \nolimits_{i = 1}^{I - 1} \mathop \sum \nolimits_{j = i + 1}^{I} \mathop \sum \nolimits_{k = 1}^{K} w_{ij} f_{ijk} }}{{\mathop \sum \nolimits_{i = 1}^{I - 1} \mathop \sum \nolimits_{j = i + 1}^{I} F_{ij} }}$$

K is the number of respondents. F_ij_ is the expected number of weighted errors in case of statistical independence (Van der Schuur 2003, pp. 148–149, 155). If there are no errors, H equals one; if the error pattern is the same as the pattern for two statistically independent variables, H equals zero. There is a simple rule of thumb to assess scale quality: a scale of items that scores lower than 0.3 should not be used as a scale (Mokken 1971).

To show that the results of these analyses are not the result of the particularity of one method, this article will also report the Cronbach’s α, a standard psychometric test of the internal consistency of items from the Classical Test Theory family (Cronbach 1951). The threshold level of α for sufficient consistency is 0.7.

### Dependent Variable and Method of Analysis

Guttman errors will be used as a measure of person-fit (Meijer 1994). They express the extent to which an individual’s responses fit on the scale. They will be used as dependent variable in this study. Guttman errors are calculated for a scale with three economic items which are listed in “Appendix 1”: an item touching on income redistribution, an item touching on economic interventionism and an item touching on the trade-off between taxes and public services.^3^

Guttman errors are count data. Count data should not be modelled with ordinary least squared regression, because the distribution is not normal and is discrete rather than continuous. For count data, Hilbe (2001) advised using either Poisson regression or negative binomial regression, depending on whether the data is overdispersed. Since the data is overdispersed (even the standard deviation is greater than the mean), negative binomial regression is used to model the number of Guttman errors. Because system-level variables were included, standard errors are clustered at the country level. Stata is employed to estimate the negative binomial regression with clustered standard errors and to calculate expected values.

### Independent Variables

To test the hypotheses, a number of independent variables were included. For the Authoritarian Legacy Hypothesis, citizens from former communist countries^4^ were given the value of one and respondents’ years of birth were used to assess the generational differences in the legacy of communist dictatorship.^5^ For reasons of interpretation, the highest value (the most recent date of birth) is one and the lowest value (the least recent date of birth) is zero. It is important to note that the interpretation of the year of birth variable is not straightforward: in a survey with only one wave, it is impossible to determine differences between age and cohort effects (Mason et al. 1973). If older respondents from Central and Eastern Europe make more Mokken errors than younger respondents from that region, this may be the result of a cohort effect, namely that these older respondents had different experiences in their youth than younger generations did. It may also result from an age effect, in that these respondents’ views on economic matters fit the left–right dimension more poorly as they grow older. One can only determine whether the citizens who were socialised during the communist period differ from those who were not; one cannot determine whether it is the combination of their year of birth and their region that causes this effect or the combination of their cohort and their region.

For the measurement of political interest, part of the Democratic Debate Hypothesis, two questions about political interest were used: whether citizens self-identify as politically interested and whether they talk about politics in their personal lives. These two items form a sufficiently strong scale (H = 0.53, Pearson’s r = 0.45). The scale was calculated so that the maximum is one and the minimum is zero.

This hypothesis also requires a measure of the extent to which the views of politicians in different countries fit the left–right dimension. To ensure that the effect is truly causal, data is necessary that predates the 2014 European Election Survey. Therefore the 2009 European Candidate Survey is employed. It queried 1576 candidate MEPs who ran for the 2009 European Parliament from 27 member states (Weßels 2011). The survey included four economic items, which are listed in “Appendix 2”. The scalability of these items was calculated in general (Table 3) and per country (Table 4). The results are discussed in detail in Sect. 5. For easier comparison in the regression, the elite-level H-values is recalculated for this analysis so that the maximum was zero and the minimum was one.

Note that the items on the 2009 survey are different from the items on the 2014 European Election Survey. This can be to the advantage of this study: rather than considering respondents’ answers on the exact same items, the measure of elite ideological consistency uses different items. If there still is a significant relationship between the number of Guttman errors politicians make and the number of Guttman errors respondents make, this indicates that voters’ views on these issues in general are affected by the way politicians talk about economic issues in general and not just the positions of the specific issues included in the survey. Moreover, the data clearly predates the voter-level data, allowing for a clearer grasp of the causal direction.

Three control variables were included in the analysis: education, class and political knowledge. As Key (1961, p. 304) argued, education in particular may ‘serve to indoctrinate people into the more-or-less official political values of the culture’. For the education variable, people who left school after the age of 20, or were still studying and older than 20, were considered to have received higher education. This is the only education-related variable in the European Election Survey. Social-economic status self-identification was used for the class variable which, according to Van der Waal et al. (2010), correlates with conformity to the left–right dimension. A distinction was made between working-class (including upper working class) and non-working-class voters. To measure respondents’ political knowledge, four different political knowledge questions were included in the survey. If a respondent indicated that they did not know the answer to a question, this response was treated as an incorrect answer.^6^ These three items barely meet the requirements for a sufficiently strong scale (H = 0.34, α = 0.52).^7^ The scale was calculated so that the maximum is one and the minimum is zero. The correlation with political interest is 0.31, which is significant but not particularly strong (Table 1).

**Table 1:** Descriptive statistics of dependent and independent variables

Variable	Mean	Median	SD	Min.	Max.	N
Guttman errors	20.19	11	22.63	0	140	25,568
Year of birth	0.42	0.42	0.21	0	1	30,064
Political interest	0.51	0.50	0.27	0	1	29,895
Political knowledge	0.71	0.75	0.27	0	1	30,064
Central and Eastern European	0.41	–	–	0	1	30,064
Education	0.37	–	–	0	1	30,064
Class	0.41	–	–	0	1	29,663
Elite-level H-value	0.43	0.47	0.21	0	1	28,986

*SD* is standard deviation, *Min* is minimum, *Max* is maximum, *N* is the number of cases

## Do Citizens’ Views on Economic Matters Fit the Left–Right Dimension?

The first question this study asked in the introduction is whether voters’ positions on economic issues fit the left–right dimension. To check this, Mokken scaling analyses on voter-level data (Table 2) were performed: an analysis of these three economic items, which are generally considered to form a single economic left–right dimension, showed a poor scalability coefficient (0.11) at the voter level. This means that the relationship between the different items is practically indistinguishable from a random pattern. As one can see from the H_ij_-values, the lack of scalability is not the result of a single poorly performing item. The value of the Cronbach’s α, also shown in Table 2, is far below the threshold. All in all, European citizens’ policy positions cannot be reduced to a single dimension.

**Table 2:** Scaling analysis (voter-level)

Variable	Interventionism	Egalitarianism	Taxes vs. services
Egalitarianism	0.17	0.04	
Taxes vs. services	0.12		
H_i_	0.14	0.11	0.08
H			0.11
Cronbach’s α			0.26

In comparison, at the elite level (Table 3), the four included items *did* form a sufficiently strong scale, both in terms of the H-values and the α-values shown. Each individual relationship was also sufficiently strong. This all indicates that—as expected—politicians’ preferences on economic matters fit the left–right dimension.

**Table 3:** Scaling Analysis (elite-level)

Variable	Private enterprise	Public services	Interventionism	Redistribution
Public services	0.51			
Interventionism	0.53	0.32		
Redistribution	0.60	0.51	0.45	
H_i_	0.55	0.45	0.43	0.52
H				0.48
Cronbach’s α				0.77

If one examines the voter-level results at the level of the individual country (Table 4), one can see mostly the same result: the H-values lie between −0.02 and 0.49. In only one country (Sweden) do citizens’ views on economic issues meet the basic requirement for scalability on both measures. The other 27 countries do not meet both thresholds.^8^ Where the values are negative (Greece and Lithuania), citizens who take a left-wing position on some issue are more likely to have right-wing positions on other issues. The α and H are very similar (Pearson’s r = 0.98; significant at the 0.01 level).

**Table 4:** Scaling analysis per country

	Voter-level		Elite-level	
Country	H-value	Cronbach’s α	H-value	Cronbach’s α
Austria	0.18	0.38	**0.73**	**0.89**
Belgium	0.13	0.30	**0.51**	**0.77**
Bulgaria	0.06	0.14	−0.06	−0.11
Croatia	0.05	0.12	–^a^	–
Cyprus	0.01	0.03	**0.41**	0.66
Czech Republic	0.05	0.14	0.29	0.57
Denmark	**0.32**	0.57	**0.61**	**0.82**
Estonia	0.06	0.16	0.28	0.57
Finland	0.25	0.49	**0.32**	0.61
France	0.19	0.39	**0.57**	**0.80**
Germany	0.14	0.32	**0.67**	**0.87**
Greece	−0.00	−0.01	**0.51**	**0.75**
Hungary	0.04	0.10	0.28	0.55
Ireland	0.10	0.24	**0.68**	**0.77**
Italy	0.13	0.29	**0.44**	**0.72**
Latvia	0.03	0.08	0.23	0.50
Lithuania	−0.02	−0.06	0.02	0.07
Luxembourg	0.07	0.19	**0.58**	**0.81**
Malta	0.16	0.36	**0.90**	**0.94**
Netherlands	0.22	0.45	**0.49**	**0.75**
Poland	0.08	0.19	**0.30**	0.58
Portugal	0.10	0.24	**0.48**	**0.70**
Romania	0.05	0.12	**0.37**	0.66
Slovakia	0.12	0.27	**0.66**	**0.85**
Slovenia	0.08	0.17	0.08	0.22
Spain	0.07	0.17	**0.48**	**0.75**
Sweden	**0.48**	**0.73**	**0.55**	**0.79**
United Kingdom	0.16	0.35	**0.45**	**0.74**

Bolded values have sufficient internal consistency

^a^Croatia did not hold European Parliament elections in 2009

There is some variance at the elite level. In most countries, elite opinion form sufficiently strong scales both in terms of the H and the α. In Bulgaria, politicians have negative H and α scores. Politicians in Lithuania, Latvia, Slovenia, the Czech Republic, Hungary and Estonia do not have economic views that can be considered a single dimension. The elites surveyed from Cyprus, Finland, Poland and Romania meet the threshold level for the H but not the α. Again, the α and H are similar (Pearson’s r = 0.93; significant at the 0.01 level).

One can also use these country-level results to get a first grasp of the expectations formulated above. The Democratic Debate Hypothesis proposed that the extent to which citizens’ views fit the left–right dimension correlates to the extent to which elites’ views fit the left–right dimension. There is indeed a significant relationship between the extent to which politicians’ and voters’ preferences fit the left–right dimension using either method (Pearson’s r = 0.43 for the H-values significant at the 0.05 level and 0.50 for the α’s significant at 0.01). The Authoritarian Legacy Hypothesis proposed that the economic views of citizens from former Central and Eastern European countries are less likely to reflect the left–right dimension than those of citizens from Western European countries are. At the country level, this expectation appears to be supported: the H and α are significantly lower in Central and Eastern Europe than in Western Europe.

## Why Do Citizens’ Views on Economic Matters Deviate from the Left–Right Dimension?

The country-level results presented above were in line with the expectations. This section will test the hypothesis with greater rigour, by analysing the number of Guttman errors citizens make, in a number of negative binomial regressions (see Table 5). Model 1 is a model without interaction terms. Model 2 only includes an interaction for the respondents’ year of birth and region of residence. Model 3 includes an interaction for a respondent’s political interest and the extent to which elites think in left–right terms about economic issues. Model 4 includes both interactions.

**Table 5:** Explaining Guttman Errors

Variable	Model 1	Model 2	Model 3	Model 4
Constant	3.04*** (0.14)	3.00*** (0.14)	2.88*** (0.13)	2.84*** (0.14)
Year of birth	−0.09 (0.06)	0.02 (0.07)	−0.08 (0.06)	0.01 (0.07)
Central and Eastern Europe	0.33*** (0.09)	0.48*** (0.12)	0.33*** (0.09)	0.46*** (0.12)
Year of birth* Central and Eastern European	–	−0.27** (0.11)	–	−0.24** (0.10)
Political interest	−0.07*** (0.03)	−0.08** (0.03)	0.25*** (0.08)	0.23*** (0.08)
Candidate-level H-value	0.01 (0.19)	0.01 (0.18)	0.33* (0.19)	0.31 (0.20)
Political interest* Elite-level H-value	–	–	−0.62*** (0.17)	−0.59*** (0.18)
Education	−0.07** (0.03)	−0.08** (0.03)	−0.07** (0.03)	−0.07** (0.03)
Political knowledge	−0.13** (0.05)	−0.13** (0.05)	−0.13** (0.05)	−0.13** (0.05)
Class	0.02 (0.02)	0.01 (0.02)	0.02 (0.02)	0.02 (0.02)
Pseudo-log likelihood	−91,287	−91,281	−91,275	−91,270
Wald Chi squared	48***	51***	56***	58***
N	22,876	22,876	22,876	22,876

Negative binomial regression with standard errors clustered by country

0.1 > * > 0.05 > ** > 0.01 > ***

**Fig. 1:**
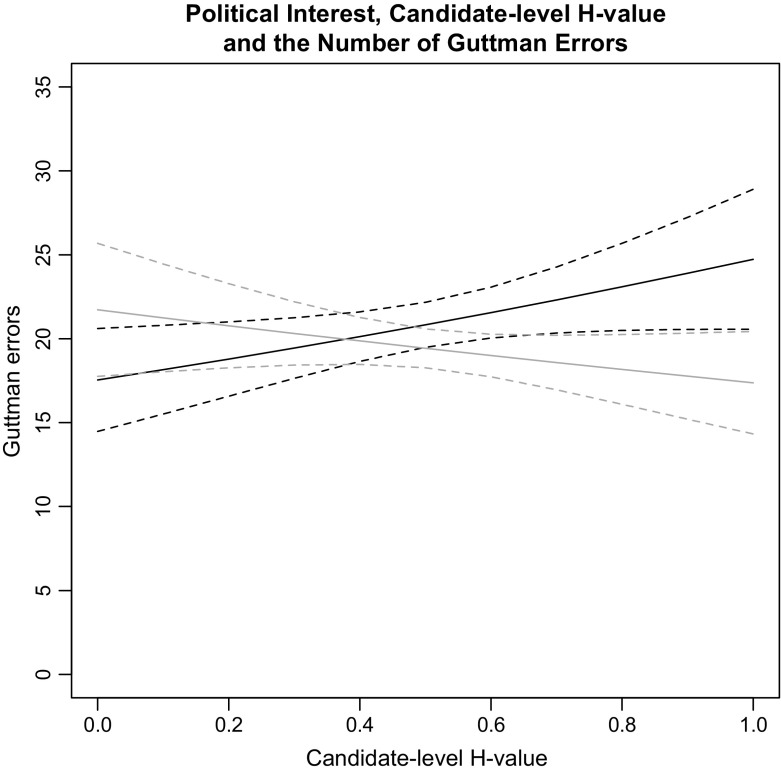
Political interest, different levels of elite consistency and the number of Guttman errors. Predicted values for Guttman errors with a 90% confidence interval for different levels of the candidate-level H-value on the horizontal dimension (which measures the extent to which politicians’ economic policy preferences correspond to the left–right model) and different levels of political interest (represented by the two lines). The grey line represents low levels of interest and the black line represents high levels of interest. Generated with the Stata margins command. Based on Model 5

First, the Democratic Debate Hypothesis is tested, which concerns the relationship between political interest and the extent to which voters and politicians hold policy positions that fit the left–right dimension. It is comprised of two parts. First, voters must pay attention to politics to receive cues from political leaders. Second, leaders must express to voters views that fit the left–right dimension. Citizens who do not pay attention to politics are less likely to have preferences that match the schemes politicians employ. Models 1 and 2 show these variables separately. They show that citizens with more political interest tend to make fewer Guttman errors. Without an interaction, there is no relationship between the extent to which elite views on economic matters fit the left–right dimension and the extent to which voters’ views do. Models 3 and 4 include this interaction. There is a significant interaction term in both models. An interaction relationship is best understood through visualisation, such as Fig. 1 (based on Model 4). There is a negative relationship between the number of errors citizens make and the extent to which politicians’ economic views fit the left–right model. When politicians’ views fit the left–right dimension, politically interested citizens make fewer errors: in the systems with the most consistent politicians, voters who pay the most attention to politics make 43% fewer errors than those who pay the least attention to politics. When politicians’ views do not fit the left–right dimension, politically interested citizens make more errors: in the systems with the least consistent politicians, voters who pay the most attention to politics make 25% more errors than those who pay no attention to politics. This difference is not significant. The difference between citizens from countries with the most and the least consistent politicians is significant beyond the halfway point of the political interest scale. Both these patterns square with the formulated expectation. Voters who pay attention to politics take cues from their leaders: when leaders cue voters to think in left–right terms, voters do so. When politicians cue voters with preferences that deviate from the left–right dimension, voters’ policy positions also deviate from this dimension.

Second, the Authoritarian Legacy Hypothesis was tested. It posited an interaction relationship between the legacy of communism and year of birth. In Models 1 and 3, respondents’ years of birth and regions are included separately. This shows that year of birth as such is not related to the number of errors made. There is a marked difference between the two regions: West European citizens are more consistent on economic issues than citizens from Central and Eastern Europe are.

**Fig. 2:**
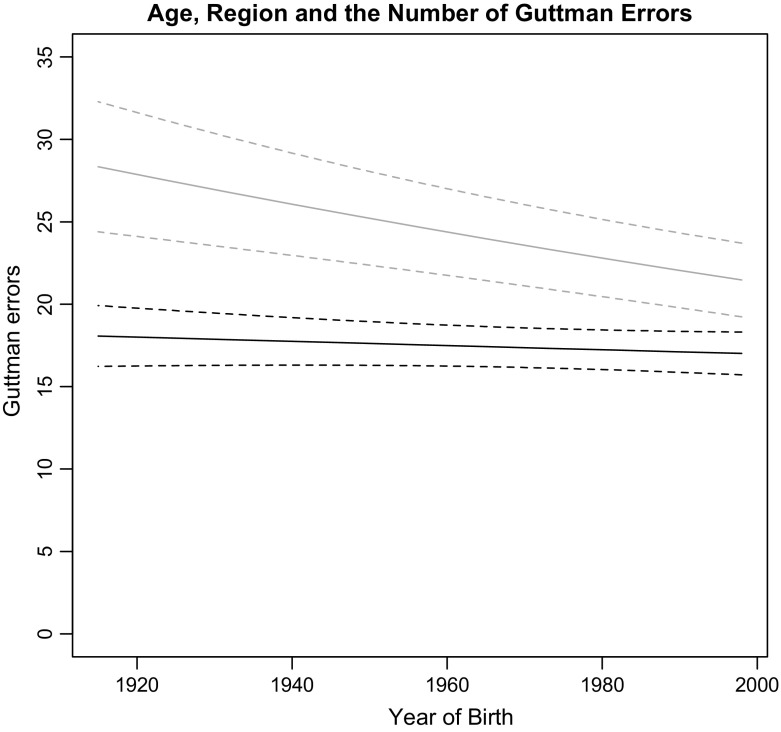
Year of birth, region and the number of Guttman errors. Predicted values for Guttman errors with a 90% confidence interval for different years of birth on the horizontal dimension and different regions (represented by the *two lines*). Generated with the Stata margins command. The *grey line* represents Central and Eastern Europe and the *black line* represents Western Europe. Based on Model 5

Models 2 and 4 include an interaction relationship, and the interaction term is significant in both models. This relationship is visualised in Fig. 2 (based on Model 4). It shows that the largest difference in the number of errors made by respondents from Western Europe compared to Central and Eastern Europe is among older people: the oldest citizens from Central and Eastern Europe made 58% more Guttman errors than their Western European contemporaries. There is still a significant difference among the youngest groups, but this has declined to 25%. This shows that there is a significant and sizeable difference between countries with and without a legacy of communism in the extent to which citizens’ views on economic matters fit the left–right dimension.

The Authoritarian Legacy Hypothesis proposed that there is an interaction relationship between the history of communism and year of birth. There is a 25% difference in the number of errors between the oldest and youngest voters in Central and Eastern Europe, compared to a 1% difference in Western Europe (not significant). As hypothesised, the economic preferences of citizens who were socialised during the communist dictatorships deviate more from the left–right dimension than the views of citizens who were socialised in the post-communist period.

The coefficients for the control variables are nearly identical in every analysis. Education and political knowledge have significant effects on the number of Guttman errors respondents make, but class does not.

All in all, the data supports both hypotheses about the causes of conformity to the left–right dimension. First, political interest contributes to *fewer* errors in systems where politicians have preferences that fit the left–right dimension, but to *more* errors in systems where politicians deviate from the left–right dimension. Second, there is a difference between Central and Eastern Europe and Western Europe in the effect of year of birth on the number of errors. These results show that one cannot just say that citizens have heterogeneous preferences.

## Conclusion

This study adds to a growing number of studies that cast doubt on the extent to which citizens’ views can be reduced to a single left–right dimension (Achterberg et al. 2011; Costello et al. 2012; De Koster et al. 2013; Derks 2004, 2006; Dolezal et al. 2013; Goerres and Prinzen 2011; Fossati and Häusermann 2014; Otjes 2014; Roosma et al. 2012, 2014; Sperber 2010; Wagner and Kritzinger 2012; Walczak et al. 2012). This study shows that the policy preferences of citizens from all over the European Union on the economic items included in the European Election Survey do not follow a unidimensional left–right pattern. This includes items that touches on economic egalitarianism, the core of the left–right dimension, according to Lipset et al. (1954), and items that touch on economic interventionism, which, according to Downs (1957), is a key element of the left–right dimension. Sufficient proof for a single economic left–right dimension among voters was found in only one of the 28 countries examined (Sweden). The results indicate that using the items studied here, a large proportion of European citizens does not have economic policy preferences that fit the standard left–right model.

The extent to which citizens’ views fit the left–right dimension was not just measurement error; it reflected differences between countries with different historical experiences and patterns of party competition and individuals from different generations and with different levels of political interest. How citizens think about economic policies reflects how politicians talk about economic policies: citizens who pay more attention to politicians whose preferences fit better to the left–right dimension tend to have policy positions that fit this dimension better. The history of communism is also reflected in the data: the preferences of those who were socialised under communist dictatorships in Central and Eastern Europe deviate the most from the economic left–right dimension. The opinions of the younger generations in Central and Eastern European fit the economic left–right dimension better than those of their elders.

This was a first attempt to delve into the extent to which voters’ opinions fit the economic left–right dimension. There may be other causes not included in this study. For instance, objective economic circumstances (e.g. unemployment) or the subjective experience of not feeling fairly treated may be more important indicators of non-conforming policy positions on economic issues (Bartels 2008). This article explained differences between Central and Eastern Europe and Western Europe by referring to the effect of alienation from politics on economic preferences in general. Perhaps the different relationship between political trust and economic interventionism differs between the Western Europe and in Central and Eastern Europe, due to different experiences with totalitarian dictatorships. A different research design would be necessary to test this relationship, one that would explain the support for interventionism as a function of the interaction between egalitarianism and political trust.

Future research may also want to delve into the effects of the fact that citizens’ views do not fit the left–right dimension but politicians’ views do, on democratic representation. If citizens’ preferences cannot be reduced to a single dimension but politicians’ views can, this implies a major challenge for representation (Van der Brug and Van Spanje 2009). In a representative democracy, elections function as instruments to link citizens’ policy preferences to the policy positions of their representatives. For this mechanism to function, a common policy dimension must structure the positions of parties and voters (Thomassen 1999). If parties only offer left-wing and right-wing policy packages but a large group of citizens want lower taxes and more government intervention, these citizens cannot find adequate representation in a party system that offers an either/or choice between these two options. They may feel unrepresented and therefore alienated from politics, undermining political trust.

## Appendix 1

See Table 5.

**Table 5:** Included items (voter level)

Item	Question	Type	Options
Interventionism	Now I would like you to tell me your views on various issues. For each issue, we will present you with two opposite statements and we will ask your opinion about these two statements. We would like to ask you to position yourself on a scale from 0 to 10, where ‘0’ means that you “0: You are fully in favour of state intervention in the economy” and ‘10’ means that you “10: You are fully opposed to state intervention in the economy”. Then if your views are somewhere in between, you can choose any number that describes your position best	Scale	11
Egalitarianism	Now I would like you to tell me your views on various issues. For each issue, we will present you with two opposite statements and we will ask your opinion about these two statements. We would like to ask you to position yourself on a scale from 0 to 10, where ‘0’ means that you “0: You are fully in favour of the redistribution of wealth from the rich to the poor in [country]” and ‘10’ means that you “10: You are fully opposed to the redistribution of wealth from the rich to the poor in [country]”. Then if your views are somewhere in between, you can choose any number that describes your position best	Scale	11
Taxes vs. services	Now I would like you to tell me your views on various issues. For each issue, we will present you with two opposite statements and we will ask your opinion about these two statements. We would like to ask you to position yourself on a scale from 0 to 10, where ‘0’ means that you “0: You are fully in favour of raising taxes to increase public services” and ‘10’ means that you “10: You are fully in favour of cutting public services to cut taxes”. Then if your views are somewhere in between, you can choose any number that describes your position best	Scale	11
Political interest	You are very interested in politics	Agreement	4
Talks about national politics	When you get together with friends or relatives, how often would you say you discuss national political matters?	Ordinal answer options	3
Knowledge 1	Switzerland is a member of the EU	True/false	2
Knowledge 2	Each member state elects the same number of representatives to the European Parliament	True/false	2
Knowledge 3	There are [150% of the correct number] members in the [lower house of the national parliament]	True/false	2
Knowledge 4	[head of government] belongs to the [correct party]	True/false	2

## Appendix 2

See Table 6.

**Table 6:** Included items (elite level)

Item	Question	Type	Options
Private enterprise	Private enterprise is the best way to solve [country’s] economic problems	Agreement	5
Public services	Public services should be in state ownership	Agreement	5
Interventionism	Politics should abstain from intervening in the economy.	Agreement	5
Egalitarianism	Income and wealth should be redistributed towards ordinary people	Agreement	5

## Footnotes

1 De Koster et al. (2013) and Van der Waal et al. (2010) also examined welfare chauvinism: voters who tend to be egalitarian do not necessarily support redistribution to immigrant groups. This is part of the new cultural dimension that Kriesi et al. (2008) argued does not play a role in the economic realm. The goal is to assess the strength of the economic left–right dimension and not to assess whether the cultural dimension also structures some aspects of economic preference.

2 Because of these weights, the number of Guttman errors can be quite high (the maximum in this analysis is 140) as stronger deviations from the expected pattern lead to increasingly high Guttman errors.

3 Costello et al. (2012) found a similar lack of conformity to the economic left–right dimension using the 2009 European Election Survey, but partially blamed the deviations from the left–right dimension on poor item wording. In 2014, the items of this survey were worded differently. The items did not ask about agreement with particular statements, but rather about citizens taking a position on particular dimensions between extremes (such as completely favouring or opposing state intervention). Therefore, these items do not suffer from acquiescence bias, which Costello et al. (2012) used to explain why citizens’ policy positions on economic issues deviated from the left–right dimension.

4 These are Bulgaria, Croatia, the Czech Republic, Estonia, Hungary, Latvia, Lithuania, Poland, Romania, Slovakia, Slovenia and the six new German *Bundesländer.*

5 The possibility of a non-linear relationship was examined by adding a quadratic term for year of birth. This did not substantially alter the results.

6 Exclusion of the respondents who did not answer the survey results in a decrease of the N of the study by 40%. Moreover, the four knowledge items do not scale in that case (H = 0.17; α = 0.33). The results are not significantly different if the cases with missing variables are excluded.

7 The low score on the α can be explained by the fact that the items were dichotomous and quite lopsided: this violates the assumptions of correlation.

8 Denmark just exceeds the threshold for the H, but falls just below the threshold for the α.
